# Missouri State Dental Association

**Published:** 1892-08

**Authors:** William Conrad

**Affiliations:** Cor. Sec'y. St. Louis, Mo.


### MISSOURI STATE DENTAL ASSOCIATION.

The twenty-seventh annual meeting of the Missouri State Dental Association was held at Clinton, Mo., July 5th to 8th inclusive. There were 26 new members admitted, and eleven papers read.

The following list of officers and committees were elected for the ensuing year:

President.—J. D. Patterson, Kansas City.
1st Vice-President —W. E. Tucker, Springfield.
2nd Vice President.—DeCoursey Lindsley, St. Louis.
Cor. Secretary.—Wm. Conrad, St. Louis.
Rec. Secretary.—H. A. Ruby, Clinton.

Treasurer.—Jas. S. Price, Weston.

Executive Committee.—C. B. Hewitt, Kansas City; A. J. McDonald, Kansas City; J. E. Crozier, Lees Summit.

Board of Censors.—E. E. Shattuck, Kansas City; H. A. Cress, Warrensburg; E. B. Crane, California.

Committee on Ethics.—Frank Slater, Rich Hill; J. B. Newby, St. Louis; C. L. Hungerford, Kansas City.

Law.—Jas. A. Price, Weston.

Committee on Publication.—J. E. Crozier, Lee's Summit; T. J. Frey, Moberly; C. L. Hickman, St. Louis.

Com. on New Appliances.—L. L. Hungerford, Kansas City.

Supervisor of Clinics.—C. H. Darby, St. Joe.

The next meeting of this Association will be held at Excelsior Springs, Mo., on the first Tuesday after July 4th, 1893.

WILLIAM CONRAD, *Cor. Sec'y.*

St. Louis, Mo.

